# Investigating white matter fibre density and morphology using fixel-based analysis

**DOI:** 10.1016/j.neuroimage.2016.09.029

**Published:** 2017-01-01

**Authors:** David A. Raffelt, J.-Donald Tournier, Robert E. Smith, David N. Vaughan, Graeme Jackson, Gerard R. Ridgway, Alan Connelly

**Affiliations:** a Florey Institute of Neuroscience and Mental Health, Melbourne, Victoria, Australia; b Department of Biomedical Engineering, Division of Imaging Sciences & Biomedical Engineering, King's College London, London, UK; c Centre for the Developing Brain, King's College London, London, UK; d Florey Department of Neuroscience and Mental Health, University of Melbourne, Melbourne, Victoria, Australia; e Department of Neurology, Austin Health and Northern Health, University of Melbourne, Melbourne, Victoria, Australia; f Department of Medicine, Austin Health and Northern Health, University of Melbourne, Melbourne, Victoria, Australia; g FMRIB Centre, Nuffield Department of Clinical Neurosciences, University of Oxford, UK; h Wellcome Trust Centre for Neuroimaging, UCL Institute of Neurology, London, UK

**Keywords:** AFD, apparent fibre density, CHARMED, composite hindered and restricted model of diffusion, CUSP-MFM, cube and sphere multi-fascicle model, DWI, diffusion-weighted imaging, FA, fractional anisotropy, FC, fibre-bundle cross-section, FD, fibre density, FDC, fibre density & cross-section, Fixel, A specific fibre population within a voxel, FBA, fixel-based analysis., FBM, fixel-based morphometry, FOD, fibre orientation distribution, FWE, family-wise error, FWHM, full width at half maximum, MRI, magnetic resonance imaging, SNR, signal-to-noise ratio, SPM, statistical parametric mapping, TBM, tensor based morphometry, VBA, voxel-based analysis., Diffusion, MRI, Fixel, Fibre, Density, Cross-section

## Abstract

Voxel-based analysis of diffusion MRI data is increasingly popular. However, most white matter voxels contain contributions from multiple fibre populations (often referred to as crossing fibres), and therefore voxel-averaged quantitative measures (e.g. fractional anisotropy) are not fibre-specific and have poor interpretability. Using higher-order diffusion models, parameters related to fibre density can be extracted for individual fibre populations within each voxel (‘*fixels*’), and recent advances in statistics enable the multi-subject analysis of such data. However, investigating within-voxel *microscopic* fibre density alone does not account for *macroscopic* differences in the white matter morphology (e.g. the calibre of a fibre bundle). In this work, we introduce a novel method to investigate the latter, which we call fixel-based morphometry (FBM). To obtain a more complete measure related to the total number of white matter axons, information from both within-voxel microscopic fibre density and macroscopic morphology must be combined. We therefore present the FBM method as an integral piece within a comprehensive fixel-based analysis framework to investigate measures of fibre density, fibre-bundle morphology (cross-section), and a combined measure of fibre density and cross-section. We performed simulations to demonstrate the proposed measures using various transformations of a numerical fibre bundle phantom. Finally, we provide an example of such an analysis by comparing a clinical patient group to a healthy control group, which demonstrates that all three measures provide distinct and complementary information. By capturing information from both sources, the combined fibre density and cross-section measure is likely to be more sensitive to certain pathologies and more directly interpretable.

## Highlights

• A fixel is defined as a specific fibre population within a voxel.
• We describe a comprehensive approach to fixel-based analysis (FBA) of white matter.
• A novel method to investigate fibre bundle morphology (cross-section) is presented.
• We compare fibre density, cross-section and a combined measure in a clinical cohort.
• The three different analyses give unique yet complementary information.

## Graphical abstract

**Figure:**
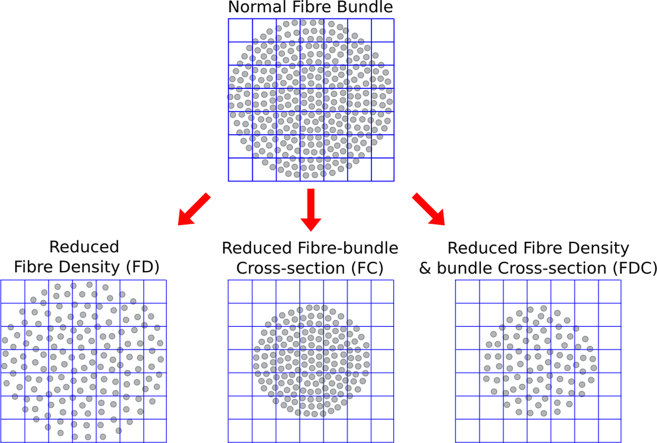

## 1 Introduction

The importance of white matter axons facilitating microsecond communication between different brain regions is evident from the severe brain dysfunction that arises in disconnection syndromes (Catani and Ffytche, 2005). Furthermore, many neurological disorders (including Motor Neurone Disease (Kassubek et al., 2012), Multiple Sclerosis (Haines et al., 2011), Epilepsy (Otte et al., 2012), and Alzheimer's disease (Radanovic et al., 2013)) involve reduction or disruption of brain ‘connectivity’ due to pathological changes to the number and density of white matter axons. In vivo methods to quantify white matter changes that alter connectivity are also of interest in relation to psychiatric disorders (Kubicki et al., 2007), development (Mills and Tamnes, 2014), aging (Lebel et al., 2012), individual differences and brain-behaviour correlations (Johansen-Berg, 2010), genetics (Meyer-Lindenberg, 2009), structural plasticity (Scholz et al., 2009), treatment response (Szeszko et al., 2014) and neuroscientific efforts to relate structural and functional connectivity (Calamante et al., 2013, Stephan et al., 2009, Van Den Heuvel et al., 2009).

Voxel-based analysis (VBA) of diffusion MRI is a common method for studying white matter, providing evidence of altered brain connectivity by detecting differences at a *local level* (Buchsbaum et al., 1998). By far the most popular approach to VBA of diffusion MRI is the analysis of diffusion tensor-derived fractional anisotropy (FA) (Basser and Pierpaoli, 1996), with voxel- or cluster-level statistical inference using packages such as SPM (http://www.fil.ion.ucl.ac.uk/spm/) or FSL (www.fmrib.ox.ac.uk/fsl). However, most white matter voxels are known to contain crossing fibres (Jeurissen et al., 2012), and voxel-averaged measures such as FA are not fibre-specific (or even erroneous) in such regions, which confounds interpretation of apparent differences (Douaud et al., 2011, Pierpaoli et al., 2001, Wheeler-Kingshott and Cercignani, 2009).

In recent years, a number of more advanced diffusion MRI models have been proposed that can resolve multiple fibre populations in a single voxel (Tournier et al., 2011). A major benefit of these so-called mixture models (Tournier et al., 2011) is that quantitative measures can be associated with a single fibre population within a voxel (Assaf and Basser, 2005, De Santis et al., 2016, Dell’Acqua et al., 2013, Raffelt et al., 2012b, Riffert et al., 2014, Scherrer et al., 2016, Scherrer and Warfield, 2012). We refer to such a single *fi*bre population within a vo*xel* as a *fixel,*^1^ as introduced in Raffelt et al. (2015). Unlike VBA, fixel-based analysis (FBA) can identify effects in specific fibre pathways even within regions containing crossing fibres (Raffelt et al., 2015),

In this work, we first discuss from a theoretical viewpoint why intra-axonal volume (which is a common quantitative measure derived from diffusion mixture models) is of biological interest in FBA of white matter. We then discuss possible mechanisms by which differences in the intra-axonal volume may manifest. This provides the basis for our assertion that when investigating intra-axonal volume, *macroscopic* white matter tract morphology should also be investigated. We therefore introduce a novel method to achieve the latter, which we call fixel-based morphometry (FBM).

The proposed FBM method provides information derived exclusively from morphology differences in fibre bundle cross-section. However, as demonstrated in our previous work (Raffelt et al., 2012b), fibre density and cross-section information can be combined to enable a more complete investigation of white matter. We therefore present the FBM method as an integral piece within a comprehensive fixel-based analysis framework to investigate measures of fibre density, fibre-bundle cross-section, and a combined measure of fibre density and bundle cross-section.

To demonstrate that FBM is appropriate for assessing fibre bundle cross-section, we performed quantitative simulations by applying a number of linear and non-linear transformations to a numerical phantom. Finally, to show how all three measures provide different yet complementary information, we include an example of a fixel-based analysis of temporal lobe epilepsy patients compared to a group of healthy control subjects.

## 2 Background

For a fixel-based analysis to be sensitive to white matter changes that affect brain ‘connectivity’, quantitative measures should ideally reflect the local white matter's ‘ability to relay information’. Many DWI models assume that diffusion within axons is restricted in the radial orientation (Alexander, 2008, Assaf and Basser, 2005, Barazany et al., 2009, Jespersen et al., 2007, Lu et al., 2006, Raffelt et al., 2012b, Stanisz et al., 1997, Zhang et al., 2012), and that the exchange of water between the intra-axonal and extra-axonal space is negligible on the timescale of a diffusion MRI experiment (Quirk et al., 2003). DWI models that estimate parameters related to the volume of intra-axonal restricted water are consequently of biological interest since this volume is influenced by the number of axons. It is therefore reasonable to consider that the intra-axonal volume (of axons within a given fixel) is a quantity related to the white matter's local ‘ability to relay information’.

In addition to the number of axons, changes in axon diameter may also influence the intra-axonal volume assigned to a given voxel or fixel. Axon diameter plays a role in the ‘ability to relay information’ via modulating transmission speed, timing and firing rate (Perge et al., 2012, Waxman, 1980). Accounting for axon diameter distributions when investigating intra-axonal volume would provide additional information and potentially more biologically meaningful metrics, however current approaches to estimate axon diameters using DWI are not able to assign estimates to individual fixels in crossing fibre regions (Alexander et al., 2010, Assaf et al., 2008). Furthermore the vast majority of axons in the human brain are smaller than 2 µm (Liewald et al., 2014), and are therefore too small to discriminate between using clinical MRI systems (Drobnjak et al., 2015).

The degree of myelination also influences white matter's capacity to transfer information. Recent work estimates fixel-specific myelin content via T1 relaxometry (De Santis et al., 2016), which would provide useful additional information when investigating fibre density. However, the current acquisition time for the required inversion recovery diffusion weighted imaging sequence is ~1 h (for whole-brain coverage), which is not suitable for most clinical populations.

### 2.1 Fibre density (FD)

In the last decade there have been numerous DWI models proposed that estimate parameters related to the “intra-axonal restricted compartment”, and the terminology employed to describe this compartment varies considerably in the literature (e.g. population fraction of the restricted compartment (Assaf and Basser, 2005), restricted fraction (De Santis et al., 2014a, De Santis et al., 2014b), axonal density (Assaf et al., 2008, De Santis et al., 2014a, De Santis et al., 2014b, Dyrby et al., 2013), partial volume fraction (Jbabdi et al., 2010), fibre density (Alexander et al., 2010, Assaf et al., 2013, Reisert et al., 2013, Riffert et al., 2014), apparent fibre density (Dell’acqua et al., 2010, Raffelt et al., 2012b), neurite density (Jespersen et al., 2010, Zhang et al., 2012), intra-axonal volume fraction (Panagiotaki et al., 2012) fibre volume fraction (Cabeen et al., 2015), fascicle fraction of occupancy (Scherrer et al., 2016)). While there are advantages and disadvantages to the different terminologies, in this work we refer to it as “fibre density” (FD) (see Section 5 for further comment on nomenclature).

**Fig. 1:**
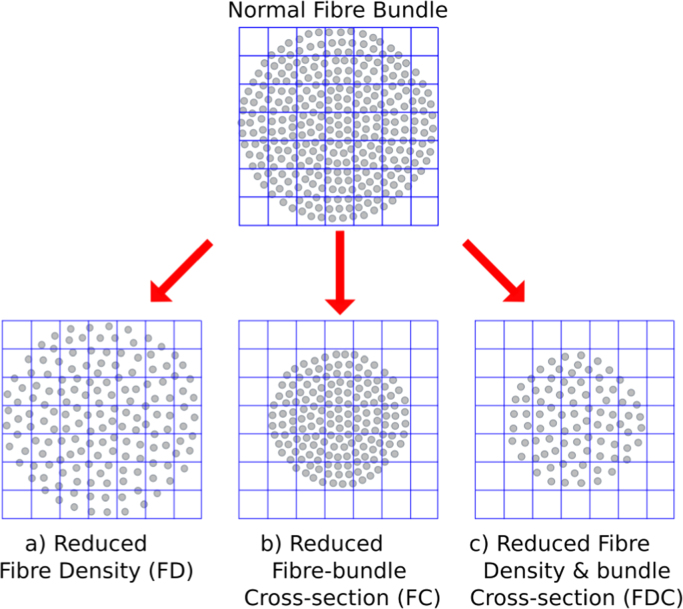
A schematic representing a fibre bundle cross-section (grey circles represent axons, while the grid represents imaging voxels). A change to the intra-axonal volume (and therefore ‘ability to relay information’) may manifest as: (a) changes in tissue *microstructure* that result in a change in within-voxel fibre density (b) a *macroscopic* difference in a fibre bundle's cross-section, or (c) a combination of both fibre density and bundle cross-sectional area.

Fig. 1 shows different ways that the intra-axonal volume of a fibre bundle may vary. Fig. 1a illustrates a reduced *volume of restricted water* within any given voxel (for example due to disease-induced axonal loss). This scenario manifests entirely as a *within-voxel* change that would be detected as a change in the diffusion-weighted signal and therefore DWI model-derived estimates of FD. While the simple schematic in Fig. 1 only depicts a single fibre bundle, we emphasise that the goal of a fixel-based analysis is to detect fibre density changes belonging to specific pathways, even in voxels containing multiple crossing fibres (Raffelt et al., 2015).

### 2.2 Fibre-bundle cross-section (FC)

Fig. 1b depicts a scenario where a difference in a fibre bundle's intra-axonal volume is manifested as a difference in the *number of voxels* the fibre bundle occupies. For example, following axon loss, the additional extra-axonal space may be persistently filled with extracellular matrix and cells related to inflammation or gliosis (as shown in Fig. 1a). However, it's also feasible that after debris are cleared, the fibre bundle becomes atrophic (white matter atrophy is a feature of many diseases including Alzheimer's disease and Motor Neurone Disease). Note that Fig. 1b would apply not only to fibre bundle differences acquired following axonal loss, but also to genetic or developmental differences in fibre bundle morphology.

Methods such as voxel-based morphometry (VBM) (Ashburner and Friston, 2000) and tensor-based morphometry (TBM) (Ashburner, 2000, Gee, 1999) have been widely used to investigate grey and white matter morphology. Both methods exploit information derived from the spatial warps computed during image registration of each subject towards a common template. At each voxel in the non-linear warp, the determinant of the Jacobian matrix (the warp's spatial derivative) describes the local differences in volume between the subject and template image. The Jacobian determinant maps can be investigated directly (TBM) or used to modulate tissue density maps (VBM).

**Fig. 2:**
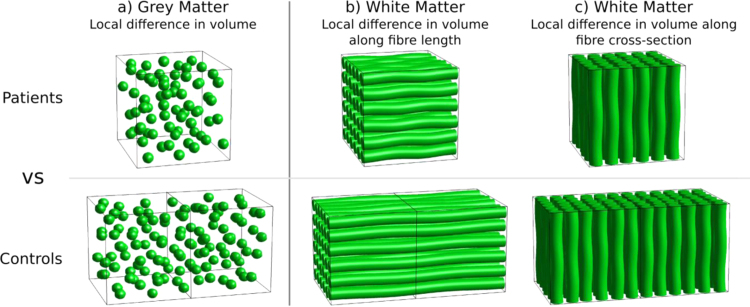
Grey matter morphometry vs white matter morphometry. (a) A local group difference in the volume of grey matter might reflect a difference in the number of neurons in that region, and therefore the Jacobian determinant is a relevant measure of interest. (b) When investigating white matter morphology, the fibre orientation must be taken into account. A local difference in the volume along the length of the fibre does not imply a difference in the number of axons. (c) A group difference in volume perpendicular to the fibre orientation (fibre-bundle cross-section) implies a difference in the number of axons and therefore the ‘ability to relay information’.

In the analysis of grey matter, changes to the number of neurons (in a local region) will likely lead to a macroscopic volumetric change (Fig. 2a). However, when investigating white matter, a difference in volume does not necessarily reflect a difference in the number of axons (and therefore ‘ability to relay information’), since the difference in volume relative to the fibre orientation is important (Raffelt et al., 2010, Zhang et al., 2009). As shown in Fig. 2b, if two groups differ locally in volume *along the length of a fibre*, then it does not imply a difference in the number of axons. In contrast, if the volume difference is *perpendicular to the fibre orientation* (i.e. a difference in fibre-bundle cross-section, FC) as shown in Fig. 2c, this implies a difference in the number of axons and therefore the ability to relay information. It is therefore essential that the fibre orientation be taken into account when investigating the morphology of white matter.

### 2.3 Fibre density and cross-section (FDC)

It is likely that in many scenarios, white matter differences may manifest as changes to both within-voxel fibre density and macroscopic fibre-bundle cross-section (Fig. 1c). Therefore, to obtain a more comprehensive measure related to the total intra-axonal volume within a pathway, both sources of information need to be taken into account and ideally be combined. Differences in a combined measure of fibre density and cross-section are more likely to reflect differences in ‘the ability to relay information’ compared to fibre density or fibre-bundle cross-section alone.

Investigating a combined measure may be particularly important for characterising diseases where neurodegeneration occurs (e.g. Alzheimer's disease or motor neuron disease), since atrophy (i.e. changes to fibre-bundle cross-section) is reflective of the accumulated axon loss, whereas within-voxel fibre density is likely to be related to the current state of the remaining white matter tissue.

The concept of combining density information with morphology information is similar in spirit to VBM (Ashburner and Friston, 2000). However, unlike DWI-derived FD metrics, T1-weighed tissue segmentations to not provide metrics that reflect cell packing density (see Section 5 for further comment).

## 3 Methods

In this section we outline the steps required to perform a comprehensive fixel-based analysis of white matter. As a critical component of this comprehensive analysis, we introduce a method called fixel-based morphometry (FBM) as a novel approach to investigate fibre-bundle cross-section (FC). We then provide quantitative simulations and an in vivo example.

### 3.1 Spatial correspondence

**Fig. 3:**
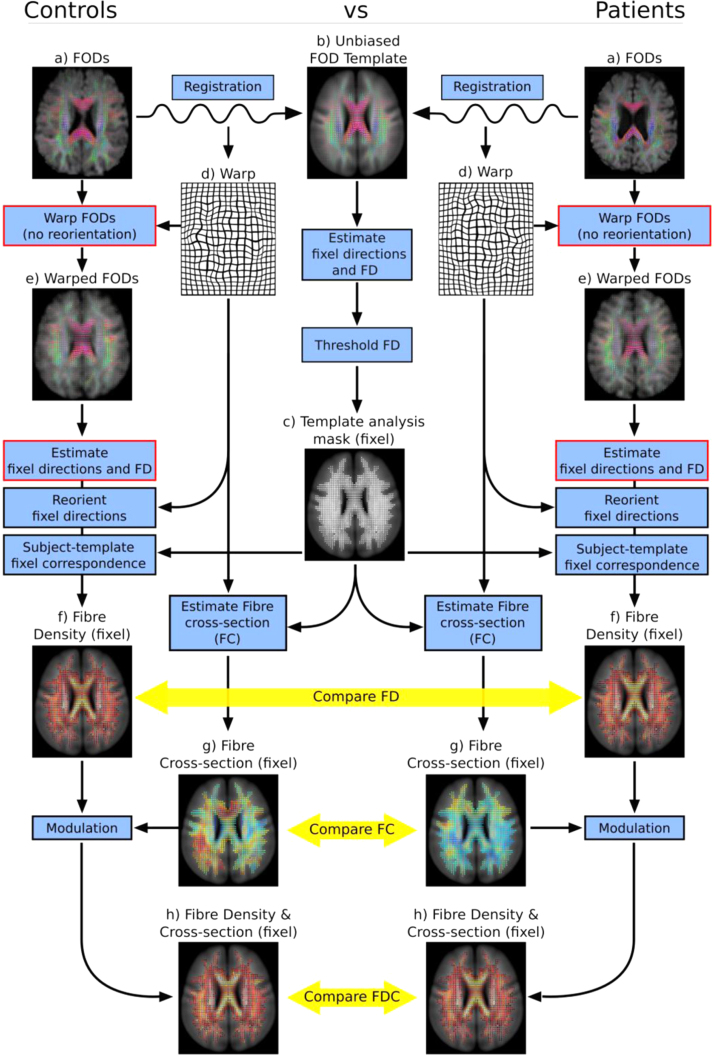
A comprehensive fixel-based analysis, illustrated for a comparison of a patient group to a control group. (a) Fibre orientation distributions (FOD) were estimated from diffusion MRI data. (b) FOD images were registered towards a study-specific group-average FOD template. (c) Each FOD in the template was segmented into individual fixels, and thresholded based on fibre density to yield a fixel-analysis mask (defining the position and orientation of all fixels-of-interest in the analysis). (d) Warps estimated from registration were used to warp FOD images to template space. (e) Each FOD in the warped images was segmented to estimate a measure of FD per fixel. Angular correspondence between subject and template fixels was obtained. (f) Fibre density was compared between groups, fixel-by-fixel. (g) As per Eq. (3), the change in fibre cross-section (w.r.t. the fixel direction), FC, was estimated from the Jacobian at each voxel in the warp, and compared between groups. (h) Fibre Density was modulated by the change in fibre-bundle cross-section to yield a combined measure of fibre density and cross-section, and compared between groups. Fixel based analysis can be performed on any fixel-based FD measure by replacing steps in red (see Section 5 for more details). (For interpretation of the references to color in this figure legend, the reader is referred to the web version of this article.)

A key step of both voxel- and fixel-based analysis is the spatial normalisation of all subject images, ideally to a representative average study-specific template (Mohammadi et al., 2012, Van Hecke et al., 2011). This involves deriving a non-linear warp for each subject that maps each point in the template image to a corresponding point in the subject image. In this work we estimated warps by registering fibre orientation distribution (FOD) images towards an unbiased study-specific FOD template (Fig. 3a and b). This was achieved by iteratively updating the template using a symmetric diffeomorphic FOD registration algorithm (Raffelt et al., 2011), which included reorientation of FODs using apodised delta functions (Raffelt et al., 2012a).

### 3.2 Fibre density

As listed in the Background section Fibre density (FD), there are several DW models that aspire to estimate quantitative measures related to fibre density. Any fixel-based measure can be employed in a fixel-based analysis; however, in this work we used apparent fibre density (AFD) (Raffelt et al., 2012b), a quantitative measure related to FD derived from FOD images computed from single-shell DWI. As described in Raffelt et al. (2012b), under certain conditions (high b-value, typical diffusion pulse duration, typical axon diameters 1–4 µm, global intensity normalisation, and a group average spherical deconvolution response function) the FOD amplitude is proportional to the intra-axonal volume of axons aligned in that direction. In this work we computed a fixel-specific measure of FD by integrating the FOD within each lobe (Fig. 3f). Briefly, FOD lobes are first segmented based on the peaks and troughs of the FOD, and the apparent FD of each lobe is calculated by non-parametric numerical integration using a dense sampling of the FOD over a hemisphere (Smith et al., 2013).

### 3.3 Angular correspondence

Following estimation of fixel FD, fixel reorientation is performed to ensure the fixel directions remain consistent with their surrounding anatomy after a non-linear spatial transformation. The new fixel orientation can be computed using Eq. (4) from Alexander et al. (2001):

(1) $${\hat{v}}_{f}^{\prime }=\frac{J{\hat{v}}_{f}}{\left\|J{\hat{v}}_{f}\right\|}$$

where ${\hat{v}}_{f}$ is the unit vector describing the original direction of the fixel, ${\hat{v}}_{f}^{\prime }$ is the reoriented direction, and J is the Jacobian matrix that describes the local affine transformation of the non-linear warp, defined as:

(2) $$J=\left[\begin{array}{ccc} \frac{\partial x'}{\partial x} & \frac{\partial x'}{\partial y} & \frac{\partial x'}{\partial z}\\ \frac{\partial y'}{\partial x} & \frac{\partial y'}{\partial y} & \frac{\partial y'}{\partial z}\\ \frac{\partial z'}{\partial x} & \frac{\partial z'}{\partial y} & \frac{\partial z'}{\partial z} \end{array}\right]$$

where ∂x′/∂x is the partial derivative of the transformation (defined as a deformation field where each template voxel contains the corresponding scanner space position of the subject image) along the x-axis with respect to dimension x in the template image. We note that performing fixel reorientation as a separate step in the processing pipeline (as opposed to performing FOD reorientation when transforming FOD images) (Fig. 3) enables any fixel-based measure of FD to be investigated within this framework (see Section 5 for more details).

To obtain correspondence of fixels across subjects we applied the method outlined in Raffelt et al. (2015). Briefly, this involves first identifying fixels of interest (i.e. a fixel template mask, Fig. 3c) by segmenting each FOD in the template image. As the template is an unbiased group average, the estimated fixels are representative of the subjects studied with respect to their locations and orientations. For each fixel in the fixel template mask, we then assign the FD value from the corresponding fixel in the subject, which is identified as the fixel with the closest orientation (within the same voxel location). As in Raffelt et al. (2015), if no subject fixel is found within a maximum angle of 30° from the template fixel (e.g. if a patient has a lesion with edema), then it is assigned a FD value of 0.

### 3.4 Fixel-based morphometry

At each point in the non-linear warp that maps template positions to the subject, information about the local scaling, shearing, and stretching is provided by the Jacobian matrix (Eq. (2)). The determinant of the Jacobian reflects local volumetric differences, where values less than one reflect shrinkage and values greater than one reflect expansion (with respect to the template). As illustrated by Fig. 2, in the analysis of white matter, volumetric changes in the plane perpendicular to the fixel orientation are of interest, since they reflect differences in the number of axons.

Here we propose to estimate a fixel-specific measure based on morphology differences in the plane perpendicular to the fixel direction, and compare this measure across subjects as a technique to investigate variation in local *fibre-bundle cross-section* (FC). More precisely, for each fixel *f* in the template fixel mask (Fig. 3c), we compute a measure that reflects the change in FC (with respect to the fixel orientation ${\hat{v}}_{f}$) required to spatially normalise the subject to the template image. This can be estimated simply as the overall volume change (Jacobian determinant), factoring out the change in scale along the direction of the fixel, giving the expansion or contraction in the perpendicular plane:

(3) $${\mathrm{FC}}_{f}=\frac{\det\left(J\right)}{\left\|J{\hat{v}}_{f}\right\|}$$

where det is the matrix determinant, ${\hat{v}}_{f}$ is the unit vector defining the direction of fixel *f*, and J is the Jacobian matrix (Eq. (2)) at the fixel's voxel location in the non-linear warp (Fig. 3d). Note that FC is estimated from the warp field that maps from template to subject space (i.e. a reverse or pull-back mapping); therefore fixel FC values >1 imply the encompassing fibre bundle has a larger cross-section in subject space, while FC values <1 imply a smaller cross-section. We note that a variation of Eq. (3) was also used in our previous work to modulate spherical harmonic point spread functions during FOD reorientation (Raffelt et al., 2012b). In Appendix A we also demonstrate that Eq. (3) is mathematically equivalent to the method used in Zhang et al. (2009) to investigate white matter morphology using the diffusion tensor.

The process of estimating a FC with respect to each template fixel is illustrated in Fig. 3c,d and g.

### 3.5 Combining fibre density and cross-section

As illustrated in Fig. 1c, group differences in the intra-axonal volume may manifest as changes to both within-voxel density and macroscopic fibre-bundle cross-section. Therefore, to obtain a more complete measure related to the total intra-axonal volume, both sources of information need to be combined.

In previous work (Raffelt et al., 2012b), we developed a method to combine both FD and FC by “modulating” Fibre Orientation Distributions (FOD) during spatial normalisation. While this method was specific to FODs that are continuous over the sphere, the same concept can be applied to any fixel-wise measure of FD. For each fixel, *f*, we compute a combined measure of fibre density and cross-section (FDC) by a multiplication (modulation) of FD by FC (Fig. 3f–h):

(4) FDC=FD×FC

As we demonstrate in the following simulations, this can be thought of as preserving the ‘total FD (i.e. intra-axonal volume)’ across the width of any bundle during a transformation. This is important for enabling direct interpretation of group differences (see the Discussion for more details).

### 3.6 Simulations using a numerical phantom

To demonstrate the FC measure (Eq. (3)) and its appropriateness for computing FDC (Eq. (4)), we applied a range of transformations (scale, shear, and non-linear warp) to a 2-dimensional numerical phantom. The phantom represents a straight fibre bundle with a simulated FD of 1 in all fixels, oriented along the x-dimension. A linear scaling was applied to alter the length of the fibre phantom (but not its width), using the transformation:

$$T_{\mathrm{scale}}=\left[\begin{array}{cc} 2 & 0\\ 0 & 1 \end{array}\right]$$

where $T_{\mathrm{scale}}$ is the conventional reverse or pull-back transformation required to map each voxel in the template to the original space. Since $T_{\mathrm{scale}}$ is a linear transformation, it was used in place of the Jacobian matrix to compute FC (Eq. (3)) and reorient the fixel direction (Eq. (1)). A shearing transformation (applied separately to the scaling) was also used, defined as:

$$T_{\mathrm{shear}}=\left[\begin{array}{cc} 1 & 0\\ 0.5 & 1 \end{array}\right]$$

To demonstrate FC under a non-linear transformation, a displacement field was simulated to both contract and expand the fibre bundle's cross-section:

$$∆y=\{\begin{array}{c} \frac{s_{x}}{8}\cos\left(\frac{2\pi x}{s_{x}}\right)\;\mathrm{if}\;y\;>\;0\\ -\frac{s_{x}}{8}\cos\left(\frac{2\pi x}{s_{x}}\right)\;\mathrm{if}\;y\;<\;0 \end{array}$$

where ∆y is the displacement of the transformation along the y-axis, $s_{x}$ is the size of the image along the x-axis (i.e. the length of the fibre), and *x* and *y* define the position of the voxel in the displacement field. To remove the discontinuity in the warp field at *y*=0 we smoothed the displacement field using a Gaussian kernel with a standard deviation of 5 voxels. Prior to estimating the Jacobian matrix, J, at each voxel position (Eq. (2)), we converted the displacement field to a deformation field (which stores the corresponding position in the transformed space as opposed to the displacement from the current voxel position). Fixel reorientation and estimation of FC was performed as in Eqs. (1), (3) respectively. In addition to the transformed FD fixel image and FC fixel image, we also computed the FDC fixel image for each transformation as per Eq. (4).

As explained in the section Fixel-based morphometry, FC is *a local measure* of the *change* in fibre cross-section that occurs during spatial normalisation. However, it is designed to detect group macroscopic differences in the fibre-bundle's cross-section (which may span several voxels). In this experiment we indirectly demonstrate that the FC measure (computed locally) is appropriate to assess group differences in fibre bundle cross-section by asserting that the sum of the FDC (which is the product of FD and FC) across the width of the fibre bundle (at any point along its length), should be equal to the sum of the FD across the width of the fibre bundle before the transformation. This is based on the assumption that the sum of the FD (or FDC) across a bundle's width is proportional to the total number of axons passing through a bundle's cross-section (and therefore related to the bundle's capacity to transfer information). If FC appropriately describes the local expansion or contraction in the plane perpendicular to the fibre orientation, then modulating FD by FC should preserve the total information carrying capacity of the bundle.

We measured the sum of the FD and FDC across the width (cross-section) of the bundle at many points along the bundle's length (Fig. 5). Because non-linear transformations may alter the shape and orientation of the fibre bundle, a fibre bundle's cross-section that defines its width may not necessarily be a linear plane. Therefore, to sum the FD and FDC across a bundle's width we performed a numerical integration by starting at the mid-line of the fibre bundle and taking sub-voxel steps (0.1) in the direction perpendicular to the interpolated fixel orientation, until we reached the bundle edge (in both directions). Results were plotted as a function of fibre bundle length.

### 3.7 In vivo example in temporal lobe epilepsy

To demonstrate how a comprehensive fixel-based analysis of FD, FC and FDC may provide unique yet complementary information, we have included an example fixel-based analysis comparing temporal lobe epilepsy patients to healthy controls.

#### 3.7.1 Participants

Patients with temporal lobe epilepsy with hippocampal sclerosis (HS-TLE: 26 patients, 13 female, 13 left epileptic focus, mean age 39.0, range 24–55 years) were compared to healthy controls (76 participants, 36 female, mean age 37.0, range 17–55 years). Hippocampal sclerosis was identified on the basis of structural MRI (Jackson et al., 1993), and the diagnosis of unilateral temporal lobe epilepsy was confirmed based on clinical assessment, video-EEG monitoring, and congruent nuclear medicine studies (FDG-PET and/or ictal SPECT). Ethical approval was obtained from the Human Research Ethics Committee of Austin Health. Written informed consent was obtained from all participants, or their parents or legal guardians in the case of minors.

#### 3.7.2 Acquisition and pre-processing

DWI was acquired on a 3T Siemens Trio (Erlangen, Germany) (60 directions at b=3000 s/mm^2^, 8 b=0 s/mm^2^, 2.5 mm isotropic). Pre-processing involved motion and bias field correction, and up-sampling by a factor of 2 (Raffelt et al., 2012b). We performed a global intensity normalisation of the DWI across subjects by dividing all volumes by the median b=0 s/mm^2^ intensity within a WM mask (Raffelt et al., 2012b). FODs were computed by robust Constrained Spherical Deconvolution (rCSD) (Tournier et al., 2013), with a group average response function (Raffelt et al., 2012b). FOD images in patients with right-sided epilepsy were flipped left-right (which included FOD reorientation (Raffelt et al., 2012a)) to align the epileptic side in all images. The same proportion (50%) of control participants were randomly selected and flipped left-right also. Spatial correspondence was obtained as described above by registering all FOD images to a symmetrical study-specific FOD template (Raffelt et al., 2011). Registration was performed using FODs at l_max_=4, 100 equally distributed apodised point spread functions during FOD reorientation, displacement field smoothing (Gaussian kernel σ^2^=1), velocity field smoothing (Gaussian kernel σ^2^=3), and an initial gradient step of 0.2.

#### 3.7.3 Fixel-based analysis

We performed a FBA of FD, FC, and FDC as summarised in Fig. 3. Measures of FD, FC and FDC were computed as described in the aforementioned sections. We compared measures of FD, FC, and FDC in all white matter fixels across both groups using a General Linear Model. For the FC and FDC analysis, we included intra-cranial volume (as computed using FreeSurfer from T1-weighted images (Dale et al., 1999)) as a nuisance covariate. To account for left-right asymmetry, we also included a nuisance covariate to indicate whether the data were flipped. Connectivity-based smoothing and statistical inference were performed with Connectivity-based Fixel Enhancement (CFE) using 2 million streamlines and default parameters (smoothing=10 mm FWHM, *C*=0.5, *E*=2, *H*=3) (Raffelt et al., 2015), where *C* is a coefficient that weights how structurally connected fixels (which are thought to share underlying axons) contribute to the enhancement of others. Similar to TFCE, the CFE *H* parameter enables a user to give more weight to extent (connected fixels) at higher test-statistic thresholds, and *E* influences how much the extent influences the enhancement as it scales in size. For further details please see Raffelt et al. (2015). Family-wise error corrected p-values were assigned to each fixel using non-parametric permutation testing (Holmes et al., 1996, Winkler et al., 2014) with 5000 permutations.

### 3.8 Visualisation of fixel-based analysis results

**Fig. 4:**
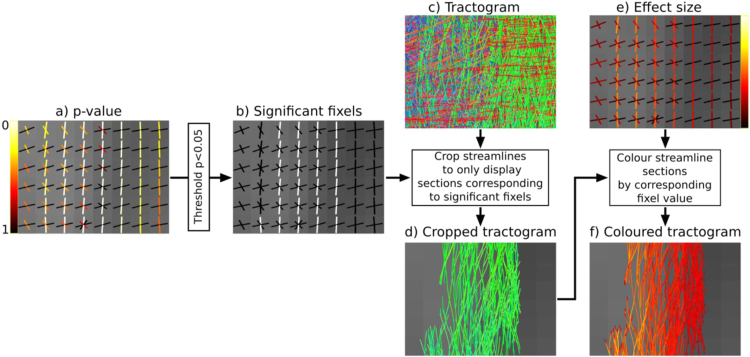
A method to visualise fixel-based analysis results using streamlines. (a) A 2D slice of fixels rendered as lines along the fibre orientation and coloured by p-value. (b) A fixel significance mask generated by thresholding p-values. (c) Whole-brain tractogram generated using the study-specific template. (d) Streamlines are cropped to display segments that correspond to significant fixels in (b) only. (e) Fixels coloured by effect size. (f) Streamlines cropped by significance and coloured by fixel effect size values in (e). (For interpretation of the references to color in this figure legend, the reader is referred to the web version of this article.)

Most white matter voxels contain multiple fixels, and therefore the results of a fixel-based analysis cannot be displayed using standard 3D image viewing software. We therefore developed a fixel overlay tool in the ‘mrview’ image viewer that is part of the MRtrix3 software package (www.mrtrix.org). This tool renders each fixel as a line drawn along the fibre orientation and colour-coded by either direction or statistic (e.g. p-value, Fig. 4a). Fixels rendered as lines are appropriate for viewing 2D slices (Fig. 4a, b and e); however to better appreciate all the fibre pathways affected and to visualise the full extent of the results in 3D, we developed a visualisation approach based on the whole-brain template-derived tractogram (Fig. 4c). We used the tractogram already computed for the aforementioned CFE statistical inference (Raffelt et al., 2015). All points within each streamline in the tractogram were assigned to an underlying fixel based on spatial location and the local streamline tangent. Streamline points were then ‘cropped’ if they corresponded to fixels that did not reach significance (p>0.05) (Fig. 4d), and the remaining points coloured by streamline orientation (left-right: red, inferior-superior: blue, anterior-posterior: green) (Fig. 4d) or fixel value of interest (e.g. p-value or effect size, Fig. 4e and f).

## 4 Results

### 4.1 Simulations on a numerical phantom

**Fig. 5:**
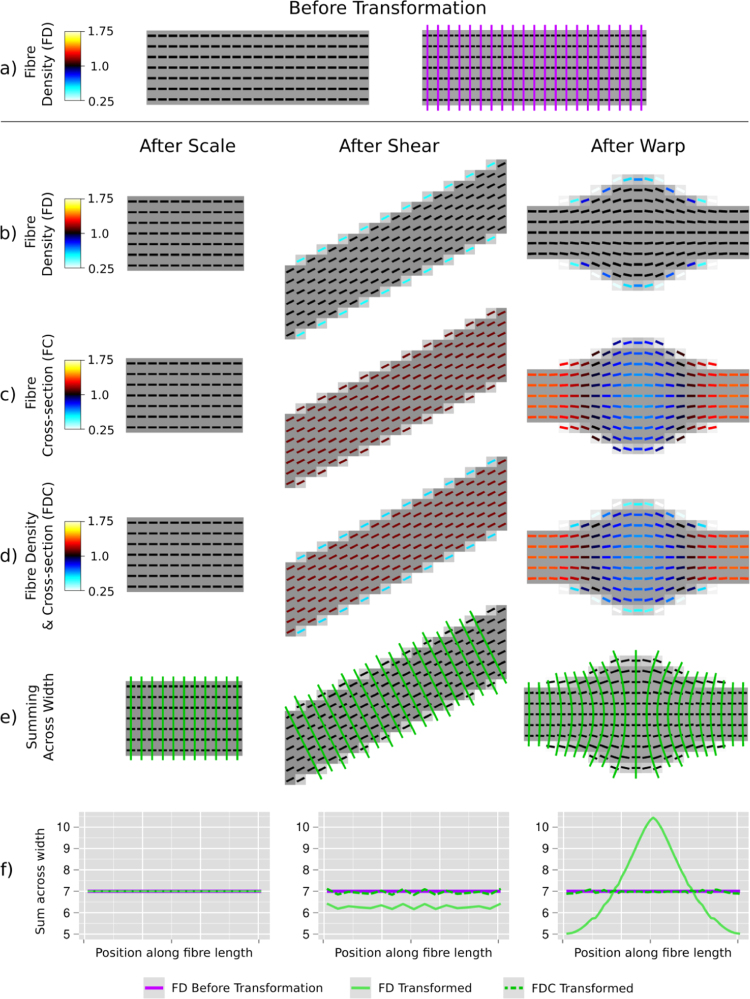
Simulations on a numerical phantom. (a) A simulated fibre bundle oriented along the x-dimension with unit FD. Shown right are cross-sections (purple) that were used to sum the FD across the width of the bundle before transformation. (b) The fibre bundle phantom after a scale, shear and non-linear warp. Fixels are colour coded by FD. (c) Transformed fibre bundles with fixels coloured by FC. (d) Transformed fibre bundles with fixels coloured by FDC. (e) Green lines indicate the cross-sections used to compute the sum of FD and the sum of FDC across the width of each fibre bundle. (f) The sum of the FD and the sum of the FDC across the width of the bundles, plotted as a function of the cross-section position along the fibre's length. (For interpretation of the references to color in this figure legend, the reader is referred to the web version of this article.)

Fig. 5 shows the results of the simulated transformations on the numerical phantom. The numerical phantom prior to transformation is shown in Fig. 5a. Fig. 5b–e shows the transformed fibre bundles with fixels colour-coded by FD, FC and FDC.

As desired, the FC measure is invariant to scale transformation since the latter only alters the fibre bundle's length, and not its width (Fig. 5c left). Therefore, the sum of the FD and the sum of FDC across the width of the bundle remain the same after the transformation (Fig. 5f left).

In the fibre bundle following a shear, the FC of all fixels is >1 indicating that the bundle width was larger prior to the shear transformation. As shown by the plot in Fig. 5f middle, because the fibre bundle is thinner after the shear, the sum of the FD across its width is less than before the transformation. However, because the FDC incorporates the change in fibre cross-section at each fixel (FC), it has the same sum across the width as before the transformation. Note that the subtle variation in the plots (as a function of position) is due to inaccuracies in the numerical integration introduced by interpolation.

As shown by Fig. 5 right, the non-linear warp changes the width of the fibre phantom, which is reflected in the FC measure at each fixel (Fig. 5c right). As shown by the plot in Fig. 5f (right), by accounting for the change in fibre cross-section, the FDC measure has the same sum across the bundle's width as the FD before the transformation.

### 4.2 Fixel-based analysis of temporal lobe epilepsy

**Fig. 6:**
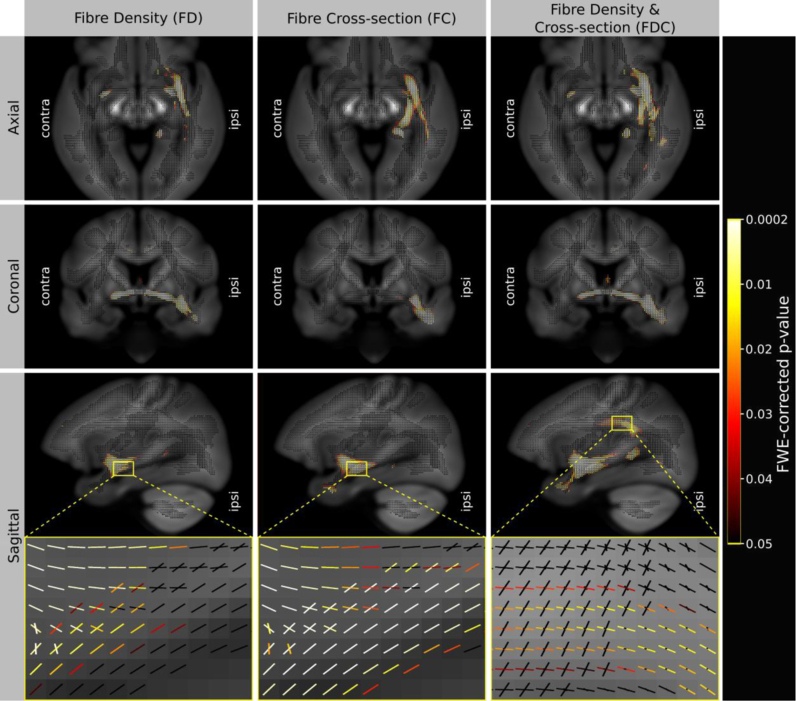
Fixels with a significant (p<0.05) decrease in fibre density (FD), fibre-bundle cross-section (FC), and fibre density and cross-section (FDC). Fixels are colour-coded by family-wise error (FWE) corrected p-values and overlaid on the total voxel-wise FD map. As shown by the zoomed in region, fixel-based analysis enables fibre tract-specific inference by attributing p-values to each fixel in voxels containing multiple fibre populations. As shown by the FDC result (right column, bottom row), combining FD and FC enabled the localisation of significant differences in additional fixels (belonging to the arcuate fasciculus). (For interpretation of the references to color in this figure legend, the reader is referred to the web version of this article.)

Shown in Fig. 6 are fixels with a significant reduction in FD, FC, and FDC in TLE compared to controls. For each view (axial, coronal and sagittal), a single 2D slice of fixels is shown, coloured by family-wise error corrected p-value and overlaid on the total voxel-wise FD map (i.e. the l=0 spherical harmonic coefficient of the FOD template). As demonstrated by the zoomed in regions (bottom row), fibre tract-specific inference is achieved by assigning an individual p-value to each fixel, rather than to each voxel.

Results suggest that TLE patients have a decrease in the number of axons that manifests as a change in both within-voxel fibre density and macroscopic fibre-bundle cross-section (Fig. 6 left and middle). As expected, group differences were maximal on the epileptic side. As shown by the FDC result (Fig. 6 right), by combining information from FD and FC additional fixels were detected as being significant (e.g. the arcuate fasciculus as shown in the sagittal view).

**Fig. 7:**
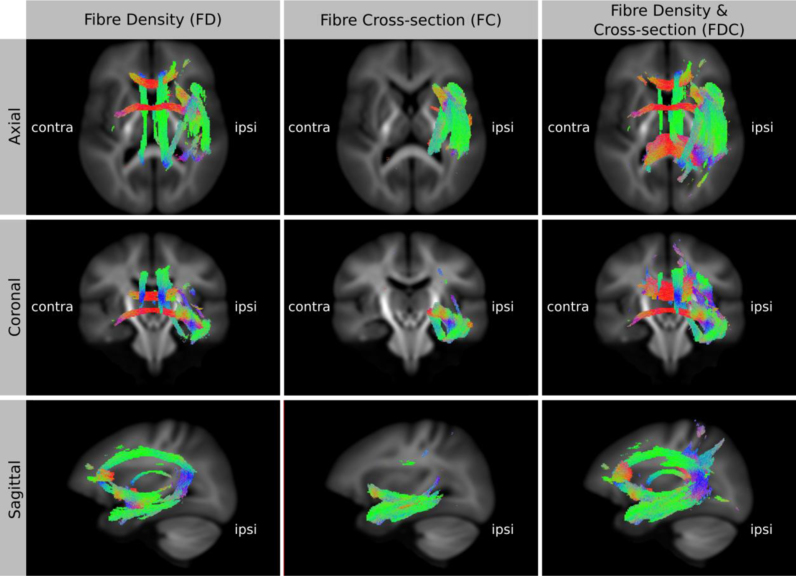
White matter pathways that have a significant decrease in FD, FC, and FDC in TLE patients compared to controls. To enable the visualisation of *all* significant fixels in 3D (i.e. not just a 2D slice), streamlines from the template-derived whole-brain tractogram were ‘cropped’ to include streamline points that correspond to significant fixels (FWE-corrected p-value <0.05), and coloured by direction (red: left-right, blue: inferior-superior, green: anterior-posterior). While there are significant group differences in both FD and FC, the combined FDC analysis detects a larger spatial extent. (For interpretation of the references to color in this figure legend, the reader is referred to the web version of this article.)

To better appreciate the extent of the group differences in 3D, and to enable a better comparison of the three different analyses (FD, FC, and FDC), we visualised the results using template-derived streamlines (as detailed in Section 3.8). Shown in Fig. 7 are streamlines that correspond to *all* white matter fixels with a significant decrease in FD, FC, and FDC, projected on top of the total voxel-wise FD map. Many of the fibre pathways that connect to the affected temporal lobe show a significant decrease in FDC. These include the cingulum, arcuate fasciculus, uncinate fasciculus, inferior frontal occipital fasciculus, fornix, anterior commissure, tapetum of corpus callosum and genu of corpus callosum. The results suggest that the main area of atrophy is located in the temporal lobe (as shown by the FC results), with reduced FD seen both in the affected temporal lobe and in tracts beyond this region. The combined measure of FDC, containing information from both FD and FC, gives the largest spatial extent of significant difference between the patient and control groups (Fig. 7).

**Fig. 8:**
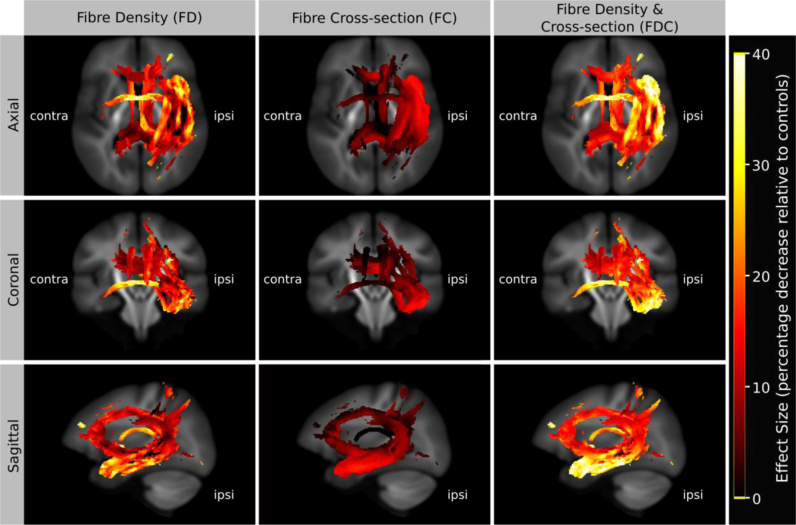
Effect sizes expressed as a percentage decrease relative to the control group. To enable a direct comparison of effect sizes across FD, FC, and FDC, streamlines shown correspond to significant fixels from all three analyses combined (i.e. the union of FD, FC, FDC). As shown left, the group differences in FD have a larger effect than FC. In both FD and FC the effect is largest in the temporal lobe. When FD is modulated by FC the effect size is increased in all pathways shown. (For interpretation of the references to color in this figure, the reader is referred to the web version of this article.)

Fig. 7 suggests that the combined FDC analysis is more sensitive because differences in both within-voxel fibre density and macroscopic fibre-bundle cross-section contribute to the measured effect. To investigate the relative effect sizes of FD and FC, and how they combine to give a larger effect size in the FDC analysis, we expressed the effect size (group difference) relative to the control group and displayed the result as colour-coded streamlines (Fig. 8). To enable a direct comparison of the effect sizes between FD, FC and FDC, we used the same streamlines to display the result from each, computed by including streamline points that correspond to significant fixels from any of the three analyses (i.e. we took the union of significant fixels (p<0.05) from FD, FC, and FDC). As shown in Fig. 8 left, TLE patients have a greater reduction in FD than FC. In both FD and FC the effect is largest in the temporal lobe. When FD is modulated by FC the effect size is increased in all pathways shown.

## 5 Discussion

### 5.1 Fixel-based morphometry

The majority of diffusion MRI analysis methods and clinical studies have focused on measures related to within-voxel microstructure only. In this work we have introduced a novel approach to white matter morphology using diffusion MRI. As explained by Fig. 1, Fig. 2, differences in fibre-bundle cross-section (FC) are of interest since they suggest a difference in the number of axons, while differences in the length of fibres should be ignored. Our numerical simulations (Fig. 5) show that the formulation of FC correctly computes the desired change in fibre cross-section, which, when combined with FD, results in a FDC measure that appropriately preserves the total FD (and therefore information carrying capacity) across a bundle's width. As demonstrated by the FC analysis of TLE patients compared to controls (Fig. 6), by accounting for the volume change with respect to each fixel's orientation, FBM enables fixel-based analysis of fibre-bundle cross-section in vivo.

Our novel FBM method has some similarities to a TBM-based approach proposed by Zhang et al. (2009), in which the Jacobian matrix is decomposed to derive a measure related to changes in the plane perpendicular to the fibre orientation. However, in that work a single diffusion tensor was used to characterise each voxel; therefore, despite the majority of white matter voxels containing crossing fibres, only a single fibre bundle is estimated in each voxel (with a potentially erroneous orientation). We also note that our method for estimating the change in fibre cross-section (FC) is mathematically equivalent to Zhang et al. (2009) (see Appendix A for proof), however it is computed in a single step and does not require the application of a Gram-Schmidt ortho-normalisation procedure.

To investigate white matter morphology, many previous studies have used VBM (Mechelli et al., 2005), which can be thought of as a TBM analysis weighted/masked by the tissue segmentation (note this is only true for modern VBM methods where images are registered as accurately as possible and modulated by the Jacobian determinant). Aside from being a voxel-based analysis, and therefore not providing fixel-specific inference, VBM obtains spatial correspondence by registering the T1-weighted images or tissue segmentations. As a consequence, registration is primarily driven by the interface of white matter with grey matter and CSF, and therefore the localisation of differences in fibre morphology within deep white matter will be strongly dependent on the registration model and regularisation parameters (for example, see Section 3.1 and Figs. 3–5 of Ashburner and Ridgway (2013)). In contrast, registration in FBM is performed using higher order DWI models, with the additional contrast improving the alignment of individual white matter bundles and thus enabling more accurate localisation of effects (the influence of the regularisation is lessened by the presence of additional information, in analogy with the influence of the prior in Bayesian analysis reducing with increasing data).

In this work we obtained spatial correspondence (and the warps used to compute FC) via registration of FOD images. While FODs benefit from high angular resolution and high b-value DWI data, FOD registration is best performed using a relatively low spherical harmonic degree of 4 (Raffelt et al., 2011). It should therefore be possible to perform FBM using FODs computed from DWI data typically acquired for diffusion tensor imaging (e.g. 20 directions and a b-value of 1000 s/mm^2^).

FBM benefits from the use of the recently developed CFE method for statistical inference (Raffelt et al., 2015). Unlike traditional cluster-based methods employed in VBM, CFE enables tract-specific smoothing and cluster-like enhancement, meaning that blurring across different structures is negligible. CFE is also less sensitive to user input parameters than other forms of cluster-based inference or cluster enhancement (Raffelt et al., 2015).

Finally, we also note that for thin white matter structures (within the scale of the voxel size), differences in fibre morphology will manifest as changes in within-voxel intra-axonal volume (i.e. FD). An example of this is shown by the absence of detected difference in the anterior commissure in the FC results shown in Fig. 6, Fig. 7. Since the anterior commissure is only a few mm wide, group differences in bundle diameter are difficult to detect at the resolution of data acquired for this study (2.5 mm isotropic). However, it is likely that a sub-voxel change to the diameter of the anterior commissure contributed to the large effect size shown in the FD results (Fig. 8 left) (see the following discussion on nomenclature), while any remaining FC effects will have a reduced statistical significance. This highlights the fact that the image resolution (and therefore partial volume effects) influences the ability to differentiate between changes in FD or FC (see below: Section 5.4). The cross-section of many white matter structures is within the spatial scale detectable by both methods (e.g. fornix, cingulum, anterior commissure), which further motivates use of the combined FDC measure to investigate FD and FC simultaneously.

### 5.2 Combining fibre density and fibre-bundle cross-section

To obtain a more complete picture of white matter morphometric effects, information from both within-voxel microscopic fibre density and macroscopic fibre-bundle cross-section can be combined. In the fixel-based analysis framework this is achieved via a simple fixel-wise multiplication of FD by FC to estimate a combined measure FDC (Eq. (4)).

The fixel-based analysis of FDC proposed here is an extension of our previous work (Raffelt et al., 2012b), where we ‘modulated’ FODs during spatial normalisation and reorientation, and performed statistical analysis on the ‘modulated FOD’ over many orientations within each voxel. However, the benefits of the currently proposed approach are that it can be applied to fixel-based measures of FD derived from any DWI model (e.g. CHARMED (Assaf and Basser, 2005)), and FDC can be analysed using superior fixel-based statistical methods (Raffelt et al., 2015).

Modulation of FD by FC is similar in concept to VBM where the total grey matter volume can be preserved during spatial normalisation by ‘modulating’ (multiplying) by the Jacobian determinant (Ashburner and Friston, 2000). However, in FBA, preserving the white matter intra-axonal volume under transformations that alter *the length of a fibre* is inappropriate, since differences in fibre length are unlikely to influence white matter's ability to relay information (Fig. 5 left). Here we modulate FD by FC (which is dependent on the fibre orientation) and therefore we preserve the intra-axonal cross-sectional area (i.e. ‘total FD’, see the following discussion on nomenclature) *across the width of a fibre bundle* during spatial normalisation to the template (Fig. 5 middle and right). Another noteworthy difference between VBM and this work is that, as pointed out in Ashburner and Friston (2000), grey matter tissue segmentations (sometimes called density or concentration maps) do not relate to the underlying cell packing density. A possible modification to VBM would be to use quantitative DWI-derived grey matter density maps (e.g. (Jeurissen et al., 2014)) instead of tissue segmentations from T1-weighted images.

In this work we have demonstrated that temporal lobe epilepsy patients have significantly reduced FD, FC and FDC in pathways that are concordant with the seizure foci (Figs. 6–8). The detection of additional significant fixels in the FDC analysis suggests an increase in sensitivity by combining FD and FC. However, we note that while FDC is likely to provide a more comprehensive assessment of white matter, FD and FC should still be investigated separately, since these may offer further insight to better characterise the effects under investigation (however see the following section on interpretation). We also note that the combined FDC analysis may not always be more sensitive if the effect of interest is predominantly in either FD or FC, since combining FD and FC also combines the variance from each source (see also the argument that modulation in VBM can increase variability, Radua et al. (2014)).

In related work, Zhang et al. (2010) also proposed a combined analysis of microscopic measures with macroscopic morphology. This was achieved by parameterising fibre bundles as 2-dimensional sheets, then projecting FA values onto each sheet (using a similar approach to Smith et al. (2006)). A morphology-based measure related to the fibre sheet thickness was estimated using DTI tractography, and co-analysed with FA using a multi-variate statistical analysis. The main limitation of this work is the parameterisation of white matter bundles using 2D sheets. While some bundles are sheet-like in shape (or at least appear to be sheet-like when tractography is based on DTI), most white matter bundles cannot be accurately modelled as a 2D sheet. Furthermore, as in TBSS (Smith et al., 2006), this approach is likely to suffer from inaccurate FA-based projection in regions of crossing fibres (Bach et al., 2014, De Groot et al., 2013, Schwarz et al., 2014), and lack of fibre-specific inference when investigating voxel-wise measures such as FA. We also note that the results from multi-variate statistics are not as directly interpretable as the univariate analysis of FDC proposed here.

### 5.3 Nomenclature

As mentioned in the background section *Fibre density*, many names have been used in previous work to describe the intra-axonal volume, each with its advantages and disadvantages. A term used in many studies is ‘density’ (Alexander et al., 2010, Assaf et al., 2013, Assaf et al., 2008; De Santis et al., 2014a, De Santis et al., 2014b; Dell’acqua et al., 2010; Dyrby et al., 2013; Jespersen et al., 2010; Raffelt et al., 2012b; Reisert et al., 2013; Riffert et al., 2014; Zhang et al., 2012). However, a problem with ‘density’ is that it may be interpreted as being solely related to a fibre bundle's number of axons *per unit area.* In the context of voxel-averaged DWI measures (e.g. Alexander et al., 2010; Zhang et al., 2012), the ‘density’ of a voxel will also be influenced by partial volume with cerebral spinal fluid. Furthermore, when referring to a fixel-specific measure (e.g. Assaf and Basser, 2005; De Santis et al., 2014a, De Santis et al., 2014b; Riffert et al. 2014; Raffelt et al., 2015), the ‘density’ of each fixel is additionally influenced by the fraction of the voxel volume occupied by other crossing fibre bundles.

Another commonly used term is ‘volume fraction’ (e.g. Cabeen et al., 2015; Jbabdi et al., 2010; Panagiotaki et al., 2012; Scherrer and Warfield, 2012). This is a more exact description, and in the context of modulation by FC, it makes more sense to preserve the sum of the ‘fibre volume fractions’ across a bundle's width compared to preserving the sum of the ‘fibre density’ (Fig. 5). However volume fraction is also not perfect. For example, “fibre or axonal volume fraction” may not be a true measure of the actual underlying volume *fractions* when the DWI model does not take into account of the different T2 of the signal arising from different compartments. Furthermore, while some multi-compartment DWI models explicitly ensure the volume fractions of all compartments sum to unity (e.g. Assaf and Basser, 2005), the apparent fibre density measure used in the present study is proportional to the measured DW signal, and hence not explicitly a volume *fraction* (Raffelt et al., 2012b). Another similar term is “intra-axonal volume”; however, this is also not ideal since it could be misinterpreted by non-technical audiences as being related to changes in individual axon volumes (i.e. their calibre). The term ‘fixel volume’ (or even ‘apparent fixel volume’ to flag that each measure has assumptions and may be dependent on experimental conditions) would be an accurate descriptor, but is too technical and far removed from the underlying biology.

In this work we opted for the term ‘fibre density’ (FD) in part because it is already common in the literature, and also since it is most easily interpretable by non-technical audiences. However, we qualify here that this should refer to the *volume of the intra-axonal compartment per unit volume of tissue* to avoid partial volume issues.

Our use of the term fibre cross-section (FC) is also not without limitations. FC may be misinterpreted as being a measurement of a particular fibre bundle's cross-section (for example something you may measure from a bundle of tractography streamlines), when it is actually measuring the *change* in fibre cross-section at the *fixel level* when undergoing spatial normalisation. Despite these issues, we believe FC is appropriate. Even if misinterpreted, the FC measure is at least related to the cross-section of the entire fibre bundle by the spatially regularised warp field (from which the Jacobian matrix is computed). It is also easily interpretable by clinical audiences and convenient when reporting results (e.g. “patients had a reduced FC in a particular fibre bundle compared to controls”).

With respect to the combined measure, FDC, we originally used the term ‘modulated (apparent) fibre density’ to describe a similar measure computed by modulated FODs (Raffelt et al., 2012b); however we believe the term FDC is more explicit with respect to the two separate sources of information and therefore more interpretable, especially alongside separate analyses of its component sources.

### 5.4 Careful interpretation of fibre density and cross-section

As depicted by Fig. 1, and demonstrated by our example analysis of TLE, differences in a fibre pathway may manifest either as a difference in within-voxel FD, a difference in macroscopic FC, or both (FDC).

Importantly, the manifestation of differences in the number of axons in a fibre pathway may change over time. For example, changes may acutely be detected as a change in FD, but over time manifest as a difference in FC (due to subsequent atrophy). In addition, as discussed above, if the degenerative white matter structure has a small cross-section with respect to the voxel size, then decreases in FC may manifest entirely as a change in FD. The issue of differences in FC being detected as FD relates to the above definition of FD being intra-axonal compartment per unit volume (i.e. partial volume). The inter-dependency of FD and FC is further understood when considering that fixels at the edge of a fibre bundle may have a smaller FD (partial volume) than those at the ‘core’ of the bundle. The proportion of ‘edge’ fixels with low FD will therefore increase as the FC decreases.

**Fig. 9:**
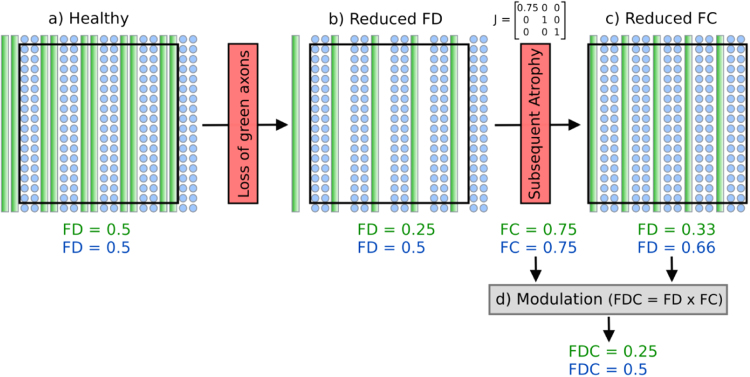
Investigating fixel FD and FC in isolation requires careful interpretation. (a) A schematic of a voxel (black square) containing two interdigitating fibre pathways with equal partial volume, crossing at 90°. (b) A scenario where half of the green fibre pathway axons have degenerated, and therefore the FD of the green fibre bundle is reduced. (c) If the remaining white matter tissue becomes atrophic as a consequence of axon loss (as indicated by a 0.75 scale in the left-right direction of the Jacobian matrix, J), then the FD of the remaining tissue now contains *an increase in the FD of the unaffected blue pathway,* while the FD of the affected green pathway has a smaller effect size. (d) By combining FD and FC, modulation ensures the atrophy is accounted for and the resulting FDC of both pathways have the expected effect size. (For interpretation of the references to color in this figure legend, the reader is referred to the web version of this article.)

The inherent inter-dependency of FD and FC highlights the need for careful interpretation when investigating fixel-based measures of FD or FC on their own. To further illustrate this, consider a hypothetical scenario where only one fibre in a crossing fibre region is affected (e.g. Douaud et al., 2011; Groeschel et al., 2014; Pierpaoli et al., 2001). Shown in Fig. 9a is a voxel that contains two crossing fibre populations with equal FD in each. If half of the axons in the green fibre were to degenerate, then one would expect an appropriate decrease in FD (Fig. 9b). However, if degeneration is subsequently followed by atrophy (i.e. contraction by 0.75 along the left-right direction) then the *within-voxel* FD for the remaining tissue now suggests an *increase* in the FD of the unaffected blue fibre bundle, and a reduced effect size of the affected green fibre bundle. If the individual differences in morphometry (FC) are not accounted for during spatial normalisation (via modulation), then the FBA results may be falsely interpreted as an increase in FD. Furthermore, Fig. 9 also highlights that differences in FC alone should also be interpreted with care. As shown, the computed FC of both fibres is the same, despite only one fibre being affected. However, we point out that the FC change in the blue fibre will only be present in the region where it crosses the atrophic green fibre, and therefore it will receive less ‘local support’ than the fixels in the green fibre in the downstream connectivity-based enhancement during statistical analysis (Raffelt et al., 2015).

As shown by Fig. 9d, when modulation is performed to estimate the combined FDC measure, the correct relative difference between the green and blue fibre is computed. This illustrates that the FDC measure may not only be more sensitive for investigating certain alterations, but also enable a more straightforward interpretation. While investigating FD and FC separately may provide biologically useful information to help understand the effects under investigation, analysis should also include the combined FDC measure to ensure the correct interpretation (Fig. 9).

### 5.5 Fixel-based analysis of other measures

In this work we derived a measure of FD from the FOD; however the proposed fixel-based analysis framework can be applied to other DWI models that aim to estimate a fixel-specific measure related to the intra-axonal volume (e.g. CHARMED (Assaf and Basser, 2005), DIAMOND (Scherrer et al., 2016)). See the following section for more details.

This work emphasises the biological relevance of DWI-derived measures related to intra-axonal volume (i.e. FD). However, we note that the proposed fixel-based analysis framework can be used to investigate other measures of interest, such as fixel-specific diffusivity measures (Scherrer et al., 2016, Scherrer and Warfield, 2012), or relaxometry (De Santis et al., 2016). We clarify that for measures that do not relate directly to fibre density, modulation of such measures by FC may not be appropriate.

### 5.6 Software availability and computation time

We have provided open-source software and step-by-step documentation on how to perform a complete fixel-based analysis (from pre-processing to the visualisation of statistical results) as part of the freely available cross-platform MRtrix3 software package (www.mrtrix.org). A complete analysis of a typical imaging cohort (<100 subjects) can be can be achieved over several days, with the most computationally expensive step being the generation of a study-specific FOD template (e.g. it takes 30 h on a 16-core server to generate a template from 20 subjects with a 1.15 mm isotropic resolution).

The analysis pipeline in MRtrix was designed to enable FBA on any fixel-based measure. This can be achieved by replacing the steps indicated by the red boxes in Fig. 3. Instead of warping FODs, DWI images can be warped (without any reorientation of the DW gradients since this is performed in a subsequent step), and instead of computing fixel directions and FD from FODs, one could estimate them from another DWI model (e.g. CHARMED).

One complication when working with fixel data is that different image voxels may have different numbers of fixels. It is therefore inefficient to store data using 4-dimensional images, since the size of the 4th dimension must accommodate the voxel with the highest number of fixels. MRtrix3 uses a custom fixel image format to handle such sparse data; however, our current work is focused on developing a more transparent format for storing fixels (i.e. directions and their values), which will utilise more common images types (e.g. NIfTI 2.0), and enable other packages to easily generate fixel data for use in MRtrix and vice versa.

## 6 Conclusion

We have delineated a framework for a comprehensive fixel-based analysis that aspires to detect differences in intra-axonal volume that manifest as differences in within-voxel fibre density and/or macroscopic fibre bundle morphology. The method handles the complex fibre-bundle configurations present in many brain voxels, and builds upon our previous work enabling tract-wise smoothing and cluster enhancement. As a core component of this analysis we have presented a novel method to investigate differences in fibre-bundle cross-section, called fixel-based morphometry, and demonstrated its applicability by identifying reduced fibre-bundle cross-section in temporal lobe epilepsy. Unlike white matter analyses using traditional voxel-based morphometry, fixel-based morphometry is fibre-specific, exploits the superior contrast provided by DWI models to drive registration, and benefits from connectivity-based statistical analysis. We therefore anticipate that FBM will prove to be a useful tool to investigate white matter morphology in future studies. Finally, we have demonstrated that by combining fibre density and cross-section, we obtain a more complete characterisation of white matter pathology that is easier to interpret than differences in fibre density or cross-section alone.

## Appendix A

Here we demonstrate that the method used for computing FC in Eq. (3) (derived independently in Raffelt et al. (2010)) is mathematically equivalent to the method for computing the change in fibre cross-section in Zhang et al. (2009). In Section 2.4 of Zhang et al. (2009) it states that:

$$\det\left(J\right)\;=\;\det\left(R\right)=s_{1}\;\times \;\det(S_{23}).$$

where $s_{1}$ is the scaling along the fibre and $\det(S_{23})$ is the change in fibre cross-section (what we define as FC). From Eq. (3) in our manuscript:

$$\det\left(J\right)=\left\|\;J{\hat{v}}_{f}\right\|\times \;{\mathrm{FC}}_{f}$$

It can be shown that $s_{1}$ is equal to $\left\|\;J{\hat{v}}_{f}\right\|$ by expanding Eq. (2) in Zhang et al. (2009):

$$\left[\begin{array}{ccc} \vdots & \vdots & \vdots \\ Je_{1} & Je_{2} & Je_{3}\\ \vdots & \vdots & \vdots \end{array}\right]=\left[\begin{array}{ccc} \vdots & \vdots & \vdots \\ \overset{´}{e_{1}} & \overset{´}{e_{2}} & \overset{´}{e_{3}}\\ \vdots & \vdots & \vdots \end{array}\right]\left[\begin{array}{cc} s_{1} & V^{T}\\ 0 & S_{23} \end{array}\right]$$

And thus:

$$Je_{1}=s_{1}\overset{´}{e_{1}}$$

From Algorithm 1 or 3 in Zhang et al. (2009):

$$\overset{´}{e_{1}}=\frac{Je_{1}}{\left\|\;Je_{1}\right\|}$$

Therefore:

$$Je_{1}=s_{1}\frac{Je_{1}}{\left\|\;Je_{1}\right\|}$$

$$\left\|\;Je_{1}\right\|=s_{1}$$

where $e_{1}$ is the vector describing the fibre orientation (what we define as ${\hat{v}}_{f}$). We note that in Zhang et al. (2009), the radial tensor eigenvectors are required unnecessarily, since the change in cross-sectional area in the plane defined by the radial eigenvectors is invariant to their orientation.

## Footnotes

1 Previous publications have used the word ‘fibre’ (Assaf and Basser, 2005), ‘fascicle’ (Rokem et al., 2015, Scherrer and Warfield, 2012) or ‘fibre population’ (Behrens et al., 2007, Raffelt et al., 2012b) to refer to a specific population of fibres within a single voxel. However, these terms can be ambiguous in certain contexts. For example, when performing statistical analysis of ‘fibres’ or ‘fascicles’, this may be misinterpreted as belonging analysis of an entire fibre pathway (e.g. a tractography-based analysis). Here, we use the word ‘fixel’ to eliminate this ambiguity when discussing fixel-specific measures and fixel-based analysis (FBA).
